# CFD-based design of air wall water blocking for underground garage entrances and exits

**DOI:** 10.1371/journal.pone.0315187

**Published:** 2025-03-19

**Authors:** Li Bin, Jiang Feng, Yan Lan, Jiang Anna

**Affiliations:** 1 Institute of Manufacturing Engineering, Huaqiao University, Xiamen, China; 2 College of Mechanical Engineering and Automation, Huaqiao University, Xiamen, China; NED University of Engineering and Technology, Pakistan

## Abstract

In the increasingly perfect underground garage construction process, the underground garage entrance water blocking problem is getting more and more attention. This paper proposes a kind of air wall water-blocking device applied to underground garage. The device is installed on the side of the straight and curved paths at the entrances and exits of the spiral underground garage. It utilizes two fans to blow the water to the drain on the other side to achieve unobstructed access to stop the water. In this paper, CFD simulation is firstly carried out on the straight road of the spiral underground garage to verify the feasibility of the program. The accuracy of the simulation results was verified by building a straight road model and conducting experiments. After that, an equal-scale 3D model of the spiral underground garage was built. Orthogonal experiments on the effects of inlet water flow velocity, fan wind speed at the straight road and fan wind speed at the curved road on the water blocking effect were carried out by using CFD simulation. A preliminary range of water velocity of 2.250 m/min to 6.786 m/min was obtained experimentally, and this range of water velocity was used as an input parameter for the simulation. The results show that the speed of water flow at the entrance of the garage and the wind speed of the fan at the straight road have a greater effect on the water blocking effect than the wind speed of the fan at the curved road. When the wind speed of the fan at the straight road is 25m/s, proper adjustment of the wind speed of the fan at the curved road can realize a good water-blocking effect of the spiral underground garage entrance within the range of water flow rate of 2.250m/min ~ 6.786m/min. Therefore, in the practical application of using the air wall water blocking scheme to realize the underground garage entrance water blocking strategy is to give priority to improve the wind speed of the fan at the straight road to improve the effect of water blocking.

## 1 Introduction

With the development of the city, the land is getting tighter and tighter, and in order to better utilize the space, underground garages are commonly promoted. However, the water barrier problem at the entrance of underground garage has been a big problem [1]. Since the entrance and exit of underground garages are usually set up with slopes, rainwater will flow into the underground garage along the slopes on rainy days, resulting in water ingress into the underground garage, and in serious cases, it will even flood the vehicles. Underground garage entrances are the main channel for rainwater to enter. Therefore the water barrier problem of underground garage entrances has been a hot issue [2].

Currently, the research for this problem is mainly divided into two ways, active water blocking and passive water blocking. Active water blocking methods include methods such as installing roll-up doors and placing rain barriers. The installation of a roller shutter door can effectively prevent rainwater from entering the underground garage, but this approach can make it difficult for vehicles to enter and exit. At the same time, the roller shutter door also has some problems, such as short service life and complex installation process [3]. Placing a rain barrier is likewise a simple and effective way of blocking water at the entrance of an underground garage. It can direct rainwater into the drain or drainage pipe, thus preventing rainwater from entering the underground garage. However, it can only be applied to small amount of rainwater and is not effective during heavy rainfall [4]. Passive water blocking methods include raising the height of underground garage entrances and installing drains. At the entrance and exit of the underground garage to do a certain height of the reverse slope [1], and set up a drainage ditch. In this way, rainwater can be blocked from entering the underground garage if the rainwater is not very large and the water on the road is not deep. During heavy rainfall, sandbags are piled up to block rainwater from entering the underground garage. Setting up drains is a more common way of blocking water at the entrance of an underground garage. But in practice, only a part of the rainwater can be channeled through the drains to the designated drainage outlets, and a large part of the rainwater flows directly into the underground garage [5]. Deng [6] suggested that in order to prevent rainwater from flowing into the underground garage, 2 or more drains should be provided for the car garage entry ramp. One of the drains is installed at the beginning of the outdoor ground ramp to stop a portion of the rainwater flowing from the outdoor ground ramp to the underground garage. The other drain is located at the end of the ramp and discharges the accumulated water through a drain pump, thus preventing the flow of rainwater into the underground garage. The underground garage air wall water barrier designed in this paper is an active water barrier. The water blocking effect of this device is better than that of passive water blocking method. Compared with the current common active water-blocking method, it can realize the dynamic adjustment of water-blocking intensity to cope with different intensity of precipitation without hindering vehicle traffic.

Currently, CFD (Computational Fluid Dynamics Simulation) technology is mainly applied to the design of ventilation systems in underground garages. Ventilation is particularly important because of the poor ventilation of underground garages, which tends to lead to high concentrations of pollutants (carbon monoxide, hydrocarbons, soot, etc.). These are categorized into mechanical and natural ventilation. In the study of mechanical ventilation in underground garages, Zhang [7] used CFD technology to numerically simulate several different forms of airflow organization design schemes to provide a good air duct design for vertical and more complex garages. Song et al. [8] calculated the ventilation volume of underground garage using two ventilation volume calculation methods, namely, the number of air changes method and the dilution concentration method, and simulated the pollutant concentration field of the underground garage under the two calculation methods using CFD simulation technology. The results show that the ventilation volume calculated by the dilution concentration method is about 52% of the ventilation volume calculated by the number of air changes method, which is favorable to the energy-saving operation of underground garage ventilation. Meng et al. [9] investigated the effects of indoor temperature, the number of air changes and the form of airflow organization on the distribution of indoor radon concentration by means of CFD numerical simulation. The results show that increasing the number of air changes can significantly reduce the indoor radon concentration. Viegas [10] also analyzed the advantages of pulsed underground garage jet fans for rapid dilution of localized pollution peaks, and Zhu et al. [11] and Papakonstantinou, K et al. [12] numerically analyzed the CO concentration in an underground garage and suggested optimization of the ventilation system. In addition, Gao and Wu [13] investigated the distribution of pollutants through numerical simulation to determine the optimal location of air purification devices. A fire in an underground garage will lead to serious consequences. Krol and Krol [14] studied the diffusion and temperature distribution of hydrogen fluoride during a small electric vehicle fire in an underground garage by using numerical analysis. Borowski et al. [15] studied the level of CO contamination, the intensity and distribution of heat and smoke during a fire, and gave recommendations on the type of jet fan as well as the location of the fan. Ren et al. [16] investigated the effect of the discharge position of the jet fan on the smoke exhaust effect using CFD simulation. The results showed that the flat jet fan would form a vortex with the fire source jet at the side wall, causing the smoke to flow back along the side wall through the vortex and gather upstream. Therefore, when adopting the method of jet fan assisted smoke exhaust, additional baffles are installed at the side wall to prevent the formation of vortex areas. Chen et al. [17] built a model of the tunnel where the water curtain system was installed. The effects of the water curtain system on smoke flow and heat propagation were investigated using CFD methods. It was found that the water curtain pressure, the number of rows, and the exothermic rate of the fire source all have an effect on the smoke blocking effect of the water curtain system. In the study of natural ventilation in underground garages, Liang et al. [18] conducted a study using CFD technology and proposed the civil construction conditions required to realize natural ventilation in underground garages. Vera et al. [19] used CFD methods to analyze air flows and temperature distribution in an underground mine working environment. The optimal ventilation system design was finally determined to improve the thermal and air quality of the underground working environment. Shen et al. [20] concluded through CFD numerical simulation that the location of the ventilation skylight has a large impact on the indoor airflow organization, and the temperature difference between indoor and outdoor also affects the thermal pressure natural ventilation. Freitas et al. [21] used CFD simulation to obtain the hydraulic pressure field acting on a dam or spillway during a flood. This method allows obtaining more accurate results than simplified analytical solutions, while being cheaper and faster to implement than physical models.

In this paper, the water blocking system at the entrance of the underground garage diverts the rainwater flowing into the underground parking garage and drains it away from the slope. Directional drainage of rainwater is achieved by a combination of air wall water blocking devices and rain grates. The program can ensure that the vehicles can still pass normally when the water-blocking system is in operation, and it has good practicability. In this paper, the effectiveness of the air wall water blocking method is verified by combining fluid dynamics simulation and physical experiment, and the optimal drainage strategy is analyzed by orthogonal experiment.

## 2 Total design and simulation verification

### 2.1 Overall design

**Fig 1:**
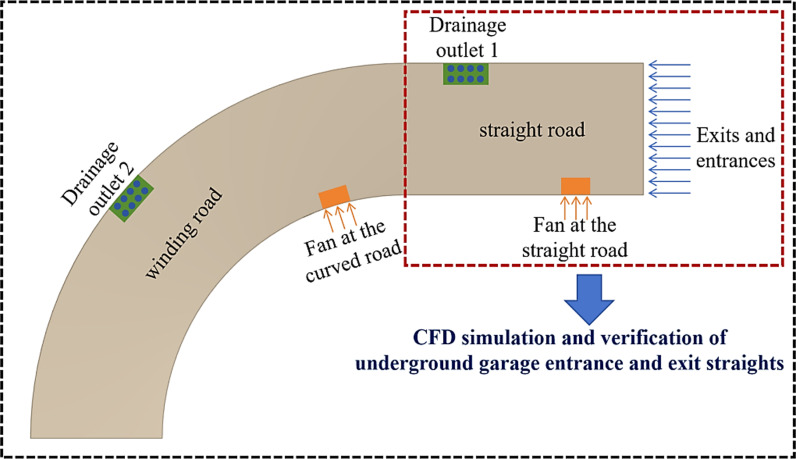
Underground garage entry/exit air wall water blocking device design plan (top view).

The design scheme of the air wall water blocking device for the underground garage is shown in Fig 1. On the straight and curved side of the driveway are fans with adjustable wind speeds. By adjusting the wind speed of the two fans, the water on the ramp will be blown to the opposite side of the drainage outlet. The main design parameters are the wind speeds of the straight and curved fans and the speed of the water flow at the garage entrance and exit.

Since the testing of the device requires the modification of the entrances and exits of the underground garage, which is costly in terms of time and modification, the design is aided by CFD simulation. In this paper, the CFD simulation of the straight part of the spiral underground garage is carried out firstly to investigate the feasibility of the program. The accuracy of the CFD simulation results is verified by building a physical model of the straight part of the spiral underground garage. Finally, an equivalent scale model of the entrance and exit of the spiral underground garage is built and CFD simulation is carried out. Orthogonal experiments are carried out to investigate the effects of water velocity, wind speed of the fan in the straight road and wind speed of the fan in the curved road on the water blocking effect. Finally, the water blocking strategy of the program in the process of practical application is concluded.

### 2.2 Fluid control equations

The governing equations of fluid mechanics include the continuity equation, the momentum equation and the energy equation. The energy equation is neglected because the temperature change in the flow process is not considered. The continuity equation can be expressed as the increment of fluid mass per unit volume of fluid per unit time is equal to the mass of fluid flowing into the volume at that time, as in Eq (1). The hydrodynamic momentum equation, also known as the Navier-Stokes equation, is expressed as the rate of change of momentum per unit volume of fluid is equal to the combined force acting on the volume [22], as in Eq (2).


$$\frac{\partial \rho }{\partial t}+\nabla \cdot \left(\rho v\right)=0$$ (1)

$$\rho \frac{dV}{dt}=\rho f-\nabla p+\mu \nabla^{2}v$$ (2)

where  ∇  is the Hamiltonian operator; *t* is the time; *ρ* is the density; *f* is the external force per unit volume of fluid; *μ* is the dynamic viscosity; and *v* is the velocity vector.

This model is a free-surface flow problem of two fluids, water and air, which are not miscible during the simulation process. Therefore, this paper adopts the VOF model in ANSYS Fluent multiphase flow model. In the VOF (volume of fluid modal) model, the interface between different phases is realized by solving the volume fraction of the fluid in each phase in the grid cell, and for the nth phase, the volume fraction equation [23] is shown in Eq (3).


$$\frac{\partial \alpha_{n}}{\partial t}+v_{n}\nabla \alpha_{n}=\frac{S_{\alpha_{n}}}{\rho_{n}}+\frac{1}{\rho_{n}}\overset{k}{\underset{n=1}{\sum }}(\vec{m_{pn}}-\vec{m_{np}})$$ (3)

where $\vec{m_{pn}}$ is the mass transfer from phase *p* to phase *n*; $\vec{m_{np}}$ is the mass transfer from phase *n* to phase *p*; and *a*_*n*_ is the volume fraction of phase n within the cell. When *a*_*n*_ = 0, it means that the nth phase is empty within the cell; when *a*_*n*_ = 1, it means that the nth phase fills the cell; and when 0 < *a*_*n*_ < 1, it means that the cell is an interface between the nth phase and other phases.

The mixed fluid density and viscosity in the cell are calculated [24] as in Eqs (4) and (5).


$$\rho \left(\vec{x},t\right)=F\left(\vec{x},t\right)\rho_{1}+\left[1-F\left(\vec{x},t\right)\right]\rho_{g}$$ (4)

$$\mu \left(\vec{x},t\right)=F\left(\vec{x},t\right)\mu_{1}+\left[1-F\left(\vec{x},t\right)\right]\mu_{g}$$ (5)

where $\rho_{g}$ is the air density; $\rho_{1}$ is the liquid water density; $\mu_{g}$ is the air viscosity; and $\mu_{1}$ is the liquid water viscosity.

The gas state at the outlet of the fan is unsteady and the flow is in a non-steady state. And this study involves the viscosity problem of the fluid near the wall. The standard *k-ε* model of turbulence model with good generalization in Fluent software is used to solve the case of this paper, and its control equations [25] are shown in Eqs (6) and (7).


$$\frac{\partial \left(\rho k\right)}{\partial t}+\frac{\partial \left(\rho ku_{i}\right)}{\partial x_{i}}=\frac{\partial }{\partial x_{j}}\left[\left(\mu +\frac{\mu_{t}}{\sigma_{k}}\right)\frac{\partial k}{\partial x_{j}}\right]+G_{k}+G_{b}-\rho \varepsilon -Y_{M}+S_{k}$$ (6)

$$\frac{\partial \left(\rho \varepsilon \right)}{\partial t}+\frac{\partial \left(\rho \varepsilon u_{i}\right)}{\partial x_{i}}=\frac{\partial }{\partial x_{j}}\left[\left(\mu +\frac{\mu_{t}}{\sigma_{\varepsilon }}\right)\frac{\partial k}{\partial x_{j}}\right]+C_{1\varepsilon }\frac{\varepsilon }{k}\left(G_{k}+C_{3\varepsilon }G_{b}\right)-C_{2\varepsilon }\rho \frac{\varepsilon^{2}}{k}+S_{\varepsilon }$$ (7)

where *G*_*k*_ is the turbulent energy term due to the laminar velocity gradient; *G*_*b*_ is the turbulent energy term due to buoyancy; and *Y*_*M*_ is the contribution term to the dissipation rate due to the expansion of the turbulent pulsations in the compressible flow to the global flow; *C*_*1ε*_, *C*_*2ε*_, *C*_*3ε*_ are three constants; *σ*_*k*_ is the k - equation turbulence Prandtl number; *σ*_*ε*_ is the ε-equation turbulence Prandtl number; *S*_*k*_ is a user-defined turbulent energy term; *S*_*ε*_ is a user-defined turbulence dissipation source term; *u*_*i*_ is the velocity of *i*-phase flow at moment *t*; *x*_*i*_ is the spatial position of the flow mass point in phase *i* at time *t*; *x*_*j*_ is the spatial position of the flow mass point in phase *j* at time *t*; *μ* is the hydrodynamic viscosity; *μ*_*t*_ is the turbulent viscosity coefficient.

In this simulation calculation, *C*_*1ε*_ is 1.44, *C*_*2ε*_ is 1.92, *σ*_*k*_ is 1.0, *σ*_*ε*_ is 1.3, and the turbulent viscosity coefficient is calculated [25] by Eq (8), where *C*_*μ*_ is the constant 0.09.


$$\mu_{t}=\rho C_{\mu }\frac{k^{2}}{\varepsilon }$$ (8)

### 2.3 Calculation working conditions and boundary conditions

In order to obtain data related to CFD analysis of gas wall water resistance and to verify the accuracy, an experimental setup was constructed for water flow velocity and flow rate measurements. The data set was used as the initial value of the simulation data as shown in Table 1.

**Table 1:** Values of flow velocity and flow rate of water.

Flow velocity u (m/min)	2.25	3.768	5.286	6.786
Flow rate *c* (L/min)	4.5	11.28	21.12	33.96

**Fig 2:**
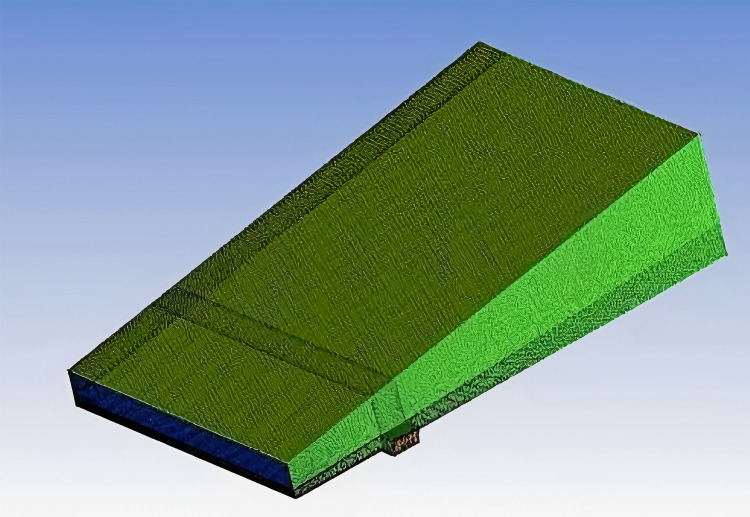
Fluid domain meshing.

The CFD software is used to mesh the fluid domain of the straight part of the spiral underground garage. The overall dimensions of the model are 9.8m in total length, 5.42m in total width, 7.8m in maximum height, and 0.297m^2^ in cross-sectional area of the wedge-shaped computational domain. The CFD mesh delineation of the fluid domain is encrypted for the air inlet, water inlet, and water outlet, etc., and the result of the mesh delineation is shown in Fig 2.

**Fig 3:**
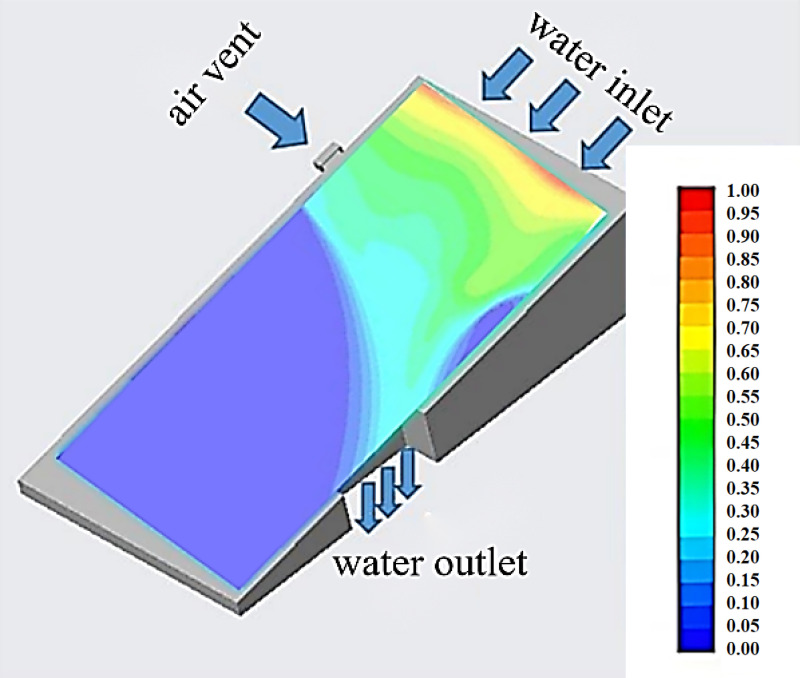
Combined simulation and 3D view.

The simulation view combined with the 3D view is shown in Fig 3. In this simulation, the water flow direction under different wind speeds was simulated by changing the wind speed magnitude. The gravitational acceleration of 9.81m/s^2^, water flow density of 998.2 kg/m^3^, water viscosity of 100.5 × 10^-5^Pa·s, air density of 1.225 kg/m^3^, and air viscosity of 1.7894 × 10^-5^Pa·s were selected.

### 2.4 Verification of CFD simulation results for straight road section

**Fig 4:**
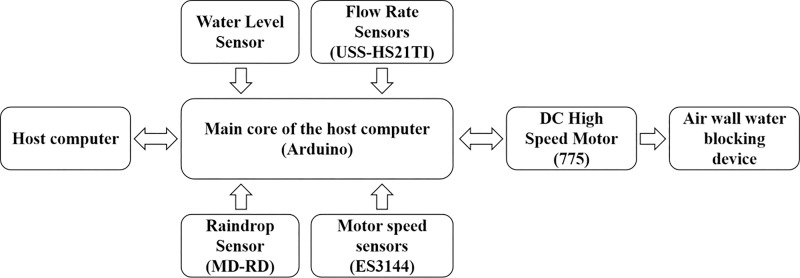
Decision-making flowchart for air-wall water-blocking devices.

The control part of the air wall water blocking device includes the upper computer, the lower computer (Arduino), and the sensors (including the water level sensor, the flow rate sensor(USS-HS21TI), the raindrop sensor(MD-RD), and the motor speed sensor(ES3144)). The control principle is to control the switch by the upper computer and monitor the working status fed back by the lower computer. When it rains, the lower computer receives and processes the signals from each sensor, and finally drives the fan and feeds back the fan (fan model 755) speed signal for dynamic adjustment. The flow chart was shown in Fig 4. The equipment that has been used in this study is commercially available

In order to obtain the actual rotational speed of the fan of the test setup in the straight road section, the simulation results of the straight road section were converted by Eqs (9)–(11), and the results are shown in Table 2.

**Table 2:** Actual speed of the fan.

*v*_*s*_ (m/s)	9	17	19	21
*n* (r/min)	10740	20280	22680	25080


$$v_{r}=\lambda \times v_{s}$$ (9)

$$v_{r}=\omega \times r$$ (10)

ω=2πn (11)

Where, *v*_*s*_ is the simulated wind speed setting value, *v*_*r*_ is the actual linear velocity of the fan, *ω* is the actual angular velocity of the fan, *n* is the actual rotational speed of the fan, the radius of the impeller *r* is taken as 0.04m, and the design parameter of the fan impeller blades, tip speed ratio *λ*, is taken as 5.

**Fig 5:**
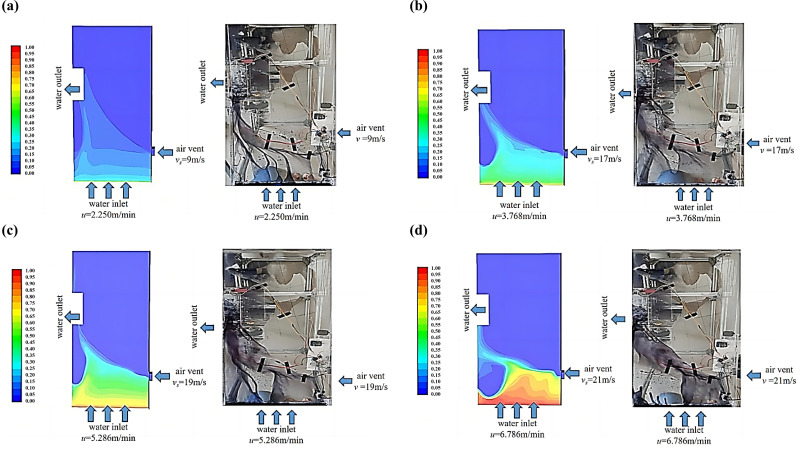
Comparison of simulation cloud view and test view. (a) The water velocity is 2.250 m/min, and the wind speed is 9 m/s. (b) The water velocity is 3.768 m/min, and the wind speed is 17 m/s. (c) The water velocity is 5.286 m/min, and the wind speed is 19 m/s. (d) The water velocity is 6.786 m/min, and the wind speed is 21 m/s.

Consistency with the CFD simulation conditions was ensured by controlling the fan speed as well as the water velocity. The simulation cloud and test views were compared as shown in Fig 5. The results are shown in Table 3.

**Table 3:** Comparison of CFD simulation results and test results.

figure number	Water flow velocity u (m/min)	Wind speed v (m/s)	Fan rotation speed *n* (r/min)	Comparative results
a	2.250	9	10000	Experimental and CFD actual values are close
b	3.768	17	20000	Experimental and CFD actual values are close
c	5.286	19	22500	Experimental and CFD actual values are close
d	6.786	21	25000	Experimental and CFD actual values are close

By comparing the CFD simulation and experimental results, it can be concluded that the water blocking effect of the straight part of the spiral underground garage is consistent with the simulation results. CFD simulation is utilized to explore the feasibility and reliability of the water blocking strategy of the spiral underground garage applying this scheme.

## 3 CFD simulation of spiral underground garage entrances and exits

### 3.1 Modeling and pre-processing

**Fig 6:**
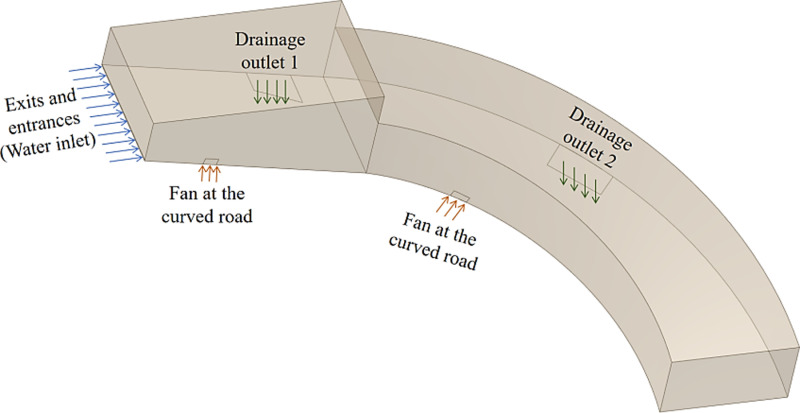
Schematic diagram of the location of the fans and drains at the entrances and exits of the spiral underground garage.

**Fig 7:**
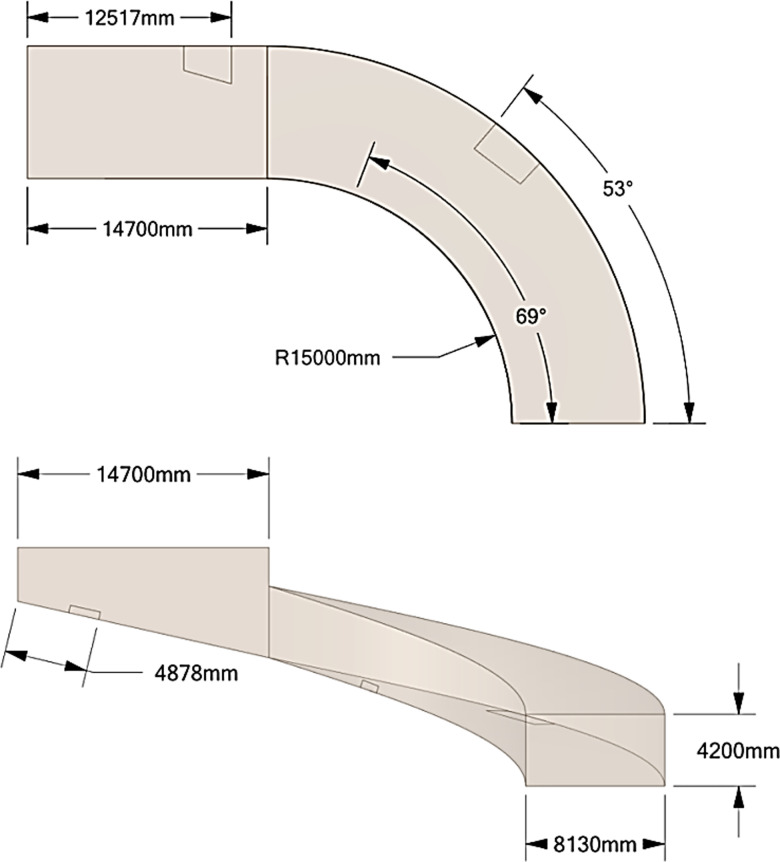
Fluid domain model.

The simulation model of the spiral underground garage entrance and exit has a width of 8.13 m and a height of 4.2 m. It is designed in accordance with the Chinese national standard “Garage Building Design Code”. The design model is an equal scale model to the actual garage, and the location of the fans and drains are shown in Fig 6. The gravitational acceleration is 9.81m/s^2^, the density of water flow is 998.2 kg/m^3^, the viscosity of water is 100.5 × 10^-5^Pa·s, the density of air is 1.225 kg/m^3^, and the viscosity of air is 1.7894 × 10^-5^Pa·s. The dimension of the fluid domain model of the spiral underground garage entrance and exit is shown in Fig 7.

**Fig 8:**
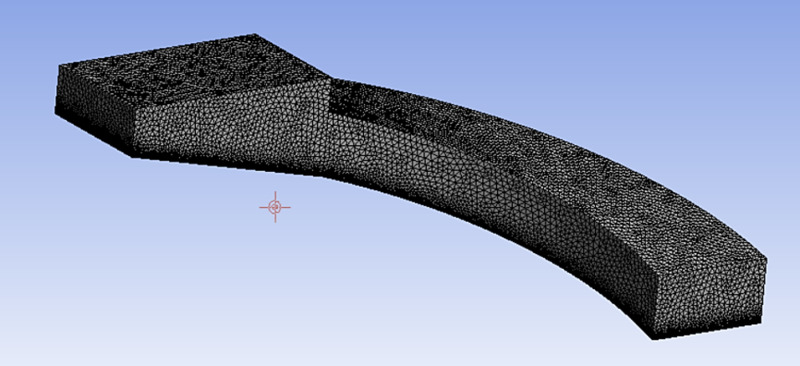
Mesh delineation of the fluid domain.

Tetrahedral mesh discretization of the computational region using ANSYS Mesh software. The simulation accuracy was improved by refining the near-surface mesh and the surface mesh of the inlet to 5 mm, and the surface mesh of the other locations to 25 mm. According to the size of the model, the maximum thickness of the expansion layer is set to 10 mm and the number of layers is 10. The minimum orthogonal mass is 0.2 and the grid quality is good to meet the requirements. The total number of meshes generated is 2.54 million, as shown in Fig 8.

### 3.2 Orthogonal experimental design

The three factors affecting the water blocking are the inlet water flow velocity and the wind speed of the fan at the straight and the fan at the curve.. The specific values of water flow velocity and fan wind speed were measured from the above experiments and assigned levels as shown in Table 4, and designed as a three-factor, four-level orthogonal experimental table as shown in Table 5. CFD simulation was carried out using ANSYS Fluent software.

**Table 4:** Rank distribution of factors.

factors	levels			
	1	2	3	4
Water velocity u (m/min)	2.250	3.768	5.286	6.786
Wind speed of the fan at the straight road *v*_1_ (m/s)	10	15	20	25
Wind speed of the fan at the curved road v_2_ (m/s)	25	20	15	10

**Table 5:** Design of orthogonal test table.

Serial number	Water velocity u (m/min)	Wind speed of the fan at the straight road *v*_1_ (m/s)	Wind speed of the fan at the curved road *v*_2_ (m/s)
1	2.250	10	25
2	2.250	15	20
3	2.250	20	15
4	2.250	25	10
5	3.768	10	20
6	3.768	15	25
7	3.768	20	10
8	3.768	25	15
9	5.286	10	15
10	5.286	15	10
11	5.286	20	25
12	5.286	25	20
13	6.786	10	10
14	6.786	15	15
15	6.786	20	20
16	6.786	25	25

The simulation results are based on whether the inlet flow is completely discharged from drain 1 and drain 2. The result is 1 if all the water is discharged from the drains, and 0 if it is not.

To verify the independence of different grid densities, we encrypted each region separately and obtained three grid quantities of 1.27 million, 2.54 million, and 5.08 million. We selected the first set of simulation parameters from Table 5 for verification. The water phase quality flow rate at outlet 1 was used as the verification object. The verification results are shown in Table 6.

**Table 6:** Results of orthogonal experiments.

	Grid 1	Grid 2	Grid 3
Number of grids	1.27 million	2.54 million	5.08 million
Mass flow rate of water phase at outlet 1	8.415 kg/s	8.423 kg/s	8.423 kg/s

The results indicate that the variation of grid density has an influence on the calculation results of less than 5%. Medium grid quantities were selected for simulation in this research.

This simulation involves water phase and air phase, so it was necessary to open the VOF model. The relevant settings of VOF were the same as those of the straight section of the underground garage entrance and exit. The turbulence model setup was the same as in the previous section, with the standard *k-ε* model selected. The water flow velocity as well as the fan air velocity in the boundary conditions were set as velocity inlets, and the specific values were set according to the orthogonal test table. The outlets were set as pressure outlets, and the pressures were standard atmospheric pressure. The simulation was an unsteady fluid simulation with a time step of 0.01s. The total simulation time was 100s.

### 3.3 Simulation results and analysis

**Fig 9:**
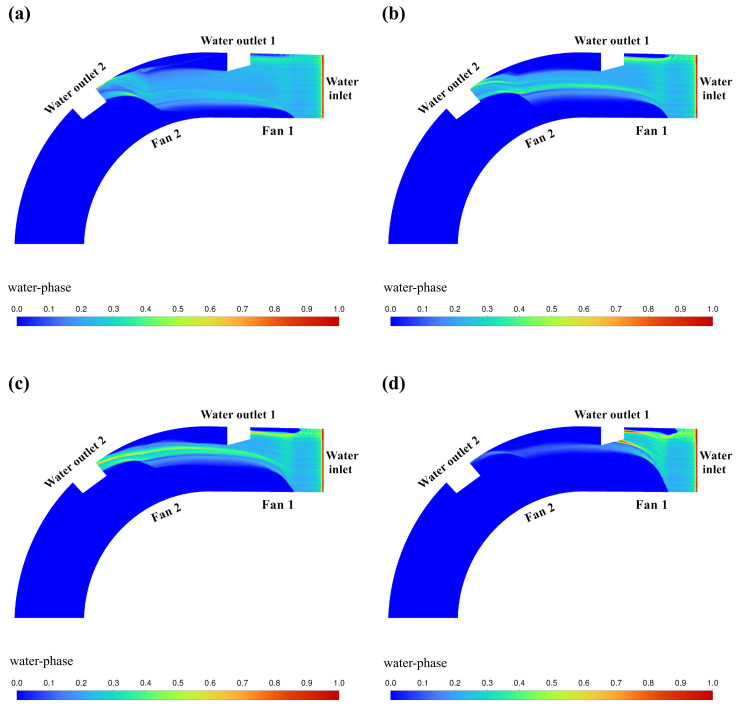
Cloud view of CFD simulation results (*u* = 2.250m/min). (a) *v*_1_ = 10m/s, *v*_2_ = 25m/s. (b) *v*_1_ = 15m/s, *v*_2_ = 20m/s. (c) *v*_1_ = 20m/s, *v*_2_ = 15m/s. (d) *v*_1_ = 25m/s, *v*_2_ = 10m/s.

The cloud of CFD simulation results based on the above orthogonal experimental scheme is shown in Fig 9–Fig 12. The results of the orthogonal experiments are shown in Table 7. Polar deviation analysis and analysis of variance (ANOVA) were carried out based on the results of the orthogonal experiments and are shown in Tables 8 and 9, respectively.

**Table 7:** Results of orthogonal experiments.

Serial number	A. Water velocity u (m/min)	B. Wind speed of the fan at the straight road. v_1_ (m/s)	C. Wind speed of the fan at the curved road. *v*_2_ (m/s)	Results
1	2.250	10	25	1
2	2.250	15	20	1
3	2.250	20	15	1
4	2.250	25	10	1
5	3.768	10	20	0
6	3.768	15	25	0
7	3.768	20	10	0
8	3.768	25	15	1
9	5.286	10	15	0
10	5.286	15	10	0
11	5.286	20	25	1
12	5.286	25	20	1
13	6.786	10	10	0
14	6.786	15	15	0
15	6.786	20	20	0
16	6.786	25	25	1

**Table 8:** Polar analysis table.

Targets		A. Water velocity u (m/min)	B. Wind speed of the fan at the straight road. v_1_ (m/s)	C. Wind speed of the fan at the curved road. *v*_2_ (m/s)
Results	K_1_	4	1	3
	K_2_	1	1	2
	K_3_	2	2	2
	K_4_	1	4	1
	R	3	3	2
	Preferred option	A_1_B_4_C_1_		

**Table 9:** Analysis of variance (ANOVA) table.

Source of variation	SS	*df*	MS	F	Significance
A. Water velocity u (m/min)	1.5	3	0.5	6	*
B. Wind speed of the fan at the straight road *v*_1_ (m/s)	1.5	3	0.5	6	*
C. Wind speed of the fan at the curved road *v*_2_ (m/s)	0.5	3	0.167	2	
*e*	0.5	6	0.083		

**Fig 10:**
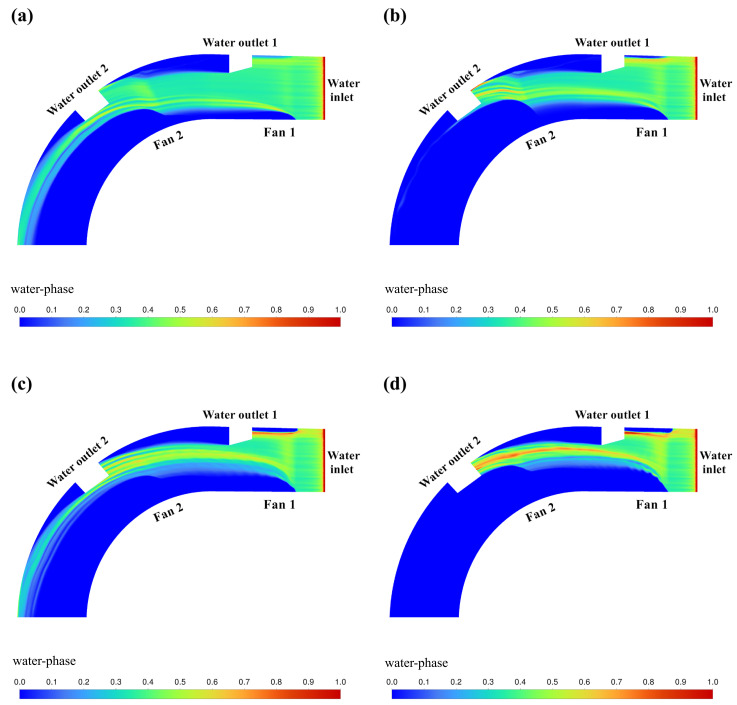
Cloud view of CFD simulation results (*u* = 3.768m/min). (a) *v*_1_ = 10m/s, *v*_2_ = 20m/s. (b) *v*_1_ = 15m/s, *v*_2_ = 25m/s. (c) *v*_1_ = 20m/s, *v*_2_ = 10m/s. (d) *v*_1_ = 25m/s, *v*_2_ = 15m/s.

**Fig 11:**
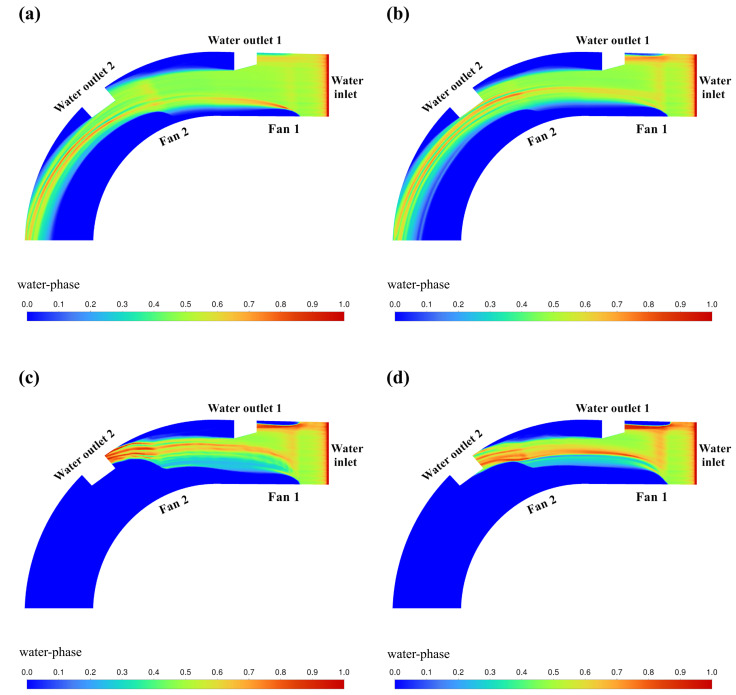
Cloud view of CFD simulation results (*u* = 5.286m/min). (a) *v*_1_ = 10m/s, *v*_2_ = 15m/s. (b) *v*_1_ = 15m/s, *v*_2_ = 10m/s. (c) *v*_1_ = 20m/s, *v*_2_ = 25m/s. (d) *v*_1_ = 25m/s, *v*_2_ = 20m/s.

**Fig 12:**
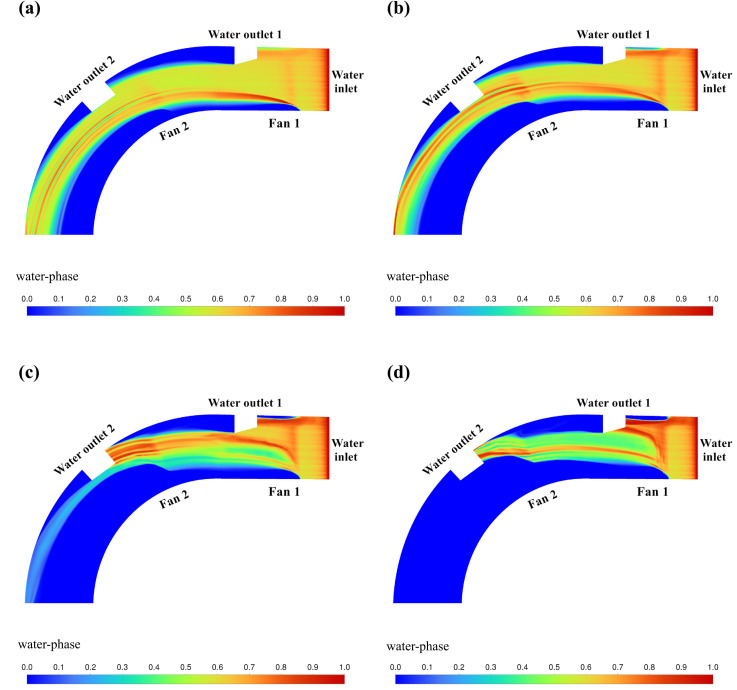
Cloud view of CFD simulation results (*u* = 6.786m/min). (a) *v*_1_ = 10m/s, *v*_2_ = 10m/s. (b) *v*_1_ = 15m/s, *v*_2_ = 15m/s. (c) *v*_1_ = 20m/s, *v*_2_ = 20m/s. (d) *v*_1_ = 25m/s, *v*_2_ = 25m/s.

Fig 9 shows the cloud image of water volume fraction simulation results obtained by setting the parameters of fan 1 (*v*_1_) and fan 2 (*v*_2_) according to the orthogonal test table when the water flow velocity *u* = 2.250m/min. Under these parameters, good water blocking effect can be achieved.

Fig 10 shows the cloud image of water volume fraction simulation results obtained by setting the parameters of fan 1 (*v*_1_) and fan 2 (*v*_2_) according to the orthogonal test table when the water flow velocity *u* = 3.768m/min. In this group of parameters, only when *v*_1_ = 25m/s, *v*_2_ = 15m/s can achieve good water blocking effect.

Fig 11 shows the cloud image of water volume fraction simulation results obtained by setting the parameters of fan 1 (*v*_1_) and fan 2 (*v*_2_) according to the orthogonal test table when the water flow velocity *u* = 5.286m/min. In this set of parameters, when *v*_1_ = 20m/s, *v*_2_ = 25m/s or *v*_1_ = 25m/s, *v*_2_ = 20m/s, good water blocking effect can be achieved.

Fig 12 shows the cloud image of water volume fraction simulation results obtained by setting the parameters of fan 1 (*v*_1_) and fan 2 (*v*_2_) according to the orthogonal test table when the water flow velocity *u* = 6.786m/min. In this group of parameters, only when *v*_1_ = 25m/s, *v*_2_ = 25m/s can achieve good water blocking effect.

The evaluation index of this experiment is that all the water flowing in from the inlet flows out from the two drains as favorable. The result of the groups that achieved good water blocking were noted as 1, otherwise were noted as 0. From the polar analysis, it can be concluded that among the three factors, the effects of water velocity (*u*) and the wind speed of the fan at the straight road (*v*_1_) on the water blocking effect are greater than that of the wind speed of the fan at the curved road (*v*_2_) on the water blocking effect. From the analysis of variance (ANOVA) and F-test, it can be concluded that the water velocity (*u*) and the wind speed of the fan at the straight road (*v*_1_) have a significant effect on water blocking, and the wind speed of the fan at the curved road (*v*_2_) has no significant effect on water blocking. Combined with polar analysis and ANOVA, it can be obtained that when the inlet water flow velocity is certain, it is prioritized to increase the wind speed of the fan at the straight road (*v*_1_) to improve the water-blocking effect.

From the simulation results, it can be concluded that when the water velocity *u* = 2.250m/min, all four wind speed levels can realize good drainage effect; when the water velocity *u* = 3.768m/min, the combination that can realize good drainage is *v*_1_ = 25m/s, *v*_2_ = 15m/s; when the water velocity *u* = 5.286m/min, the combinations that can achieve good drainage are *v*_1_ = 20m/s, *v*_2_ = 25m/s and *v*_1_ = 25m/s, *v*_2_ = 20m/s; when the water velocity *u* = 6.786m/min, the combination that can realize good drainage is *v*_1_ = 25m/s, *v*_2_ = 25m/s. By analyzing the combination that can achieve good water-blocking effect, it can be concluded that when the wind speed of the fan at the straight road is 25m/s, the wind speed of fan at the curved road can be adjusted appropriately to achieve a good water-blocking effect of the spiral underground garage entrance and exit within the range of water velocity of 2.250m/min ~ 6.786m/min. In addition, when the water velocity is certain, by comparing the experimental groups that have exchanged the values of the wind speeds of the fan at the straight road and the fan at the curved road, it can be concluded that the water-blocking effect when the wind speed of the fan at the straight road is greater than the wind speed of the fan at the curved road is better than the water-blocking effect when the wind speed of the fan at the curved road is greater than the wind speed of the fan at the straight road. In summary, since the rainfall in the actual situation is constantly changing, the strategy to realize water blocking at the entrance and exit of the underground garage by using the air wall water blocking scheme in practice is to prioritize the increase of the wind speed of the straight-road fan to improve the efficiency of water blocking.

## 4 Conclusions

In this paper, an air wall water blocking device was designed to address the problem of water blocking at the entrances and exits of spiral underground garages. The device was installed on the side of the entrance and exit of the spiral underground garage, and the fans were utilized to blow the water to the other side for discharge. It realizes water blocking from the source without obstructing the passage. In this paper, the CFD simulation of the straight road section of the spiral underground garage is firstly carried out to explore the feasibility of the scheme. Secondly, the accuracy of the scheme using CFD simulation is validated by building a physical model of the straight road part of the spiral underground garage. Finally, an equal scale model of the entrances and exits of the spiral underground garage was built and orthogonal experiments were conducted using CFD simulation. Through the polar analysis, ANOVA analysis and the simulation result cloud figures, it is concluded that the speed of water flow and the wind speed of the fan at the straight road have a greater influence on the water blocking effect of the spiral underground garage than that of the wind speed of the fan at the curved road on the water blocking effect. When the wind speed of the fan at the straight road is 25m/s, the wind speed of the fan at the curved road can realize the good water blocking effect of the spiral underground garage entrance/exit with the water velocity ranging from 2.250m/min to 6.786m/min through appropriate adjustment. The air wall water blocking program in the actual application of the process to give priority to improve the wind speed of the fan at the straight road as the main, improve the wind speed of the fan at the curved road as a complementary strategy to improve the effect of spiral underground garage water blocking. According to the simulation results in this paper, when the flow rate *u*>  2.250m/min, the fan parameters should be set to *v*_1_ ≥ 25m/s, and then adjust the v2 parameters. The limitation of this paper is the relative brevity of the trials. The next step in the process is to conduct an equal-scale physical test. This physical test component is currently being actively pursued. The advantage of the air wall water blocking system over other water blocking solutions is that it does not affect the normal passage of the channel. It can be widely used in underground garage entrances and exits to ensure the normal travel of vehicles and block rainwater into the underground garage. Further research can discuss the installation position of the air wall water blocking system, including the spacing of the fans, ground clearance and installation angle and other factors affecting the water blocking effect. Another direction of this research is to realize the dynamic regulation and distribution of fan power through multi-sensor fusion, which ultimately achieves the goal of green energy saving.
